# Risk factors for *Plasmodium falciparum* infection in the Kenyan Highlands: a cohort study

**DOI:** 10.1093/trstmh/try122

**Published:** 2018-11-29

**Authors:** Jackie Cook, Chrispin Owaga, Elizabeth Marube, Amrish Baidjoe, Gillian Stresman, Robin Migiro, Jon Cox, Chris Drakeley, Jennifer C Stevenson

**Affiliations:** 1 London School of Hygiene and Tropical Medicine, Keppel Street, London, UK; 2 Evidence Action, Ngong Road, Nairobi, Kenya; 3 Kenya Medical Research Institute (KEMRI), KEMRI-Wellcome Trust Research Programme, Kemri Square, Kilifi, Kenya; 4 Imperial College London, Praed Street, London, UK; 5 Macha Research Trust, Choma, Southern Province, Zambia; 6 Johns Hopkins Malaria Research Institute, Bloomberg School of Public Health, Baltimore, USA

**Keywords:** cohort, heterogeneity, highlands, Kenya, malaria, *Plasmodium falciparum*

## Abstract

**Background:**

Malaria transmission in African highland areas can be prone to epidemics, with minor fluctuations in temperature or altitude resulting in highly heterogeneous transmission. In the Kenyan Highlands, where malaria prevalence has been increasing, characterising malaria incidence and identifying risk factors for infection is complicated by asymptomatic infection.

**Methods:**

This all-age cohort study, one element of the Malaria Transmission Consortium, involved monthly follow-up of 3155 residents of the Kisii and Rachuonyo South districts during June 2009–June 2010. Participants were tested for malaria using rapid diagnostic testing at every visit, regardless of symptoms.

**Results:**

The incidence of *Plasmodium falciparum* infection was 0.2 cases per person, although infections were clustered within individuals and over time, with the majority of infections detected in the last month of the cohort study. Overall, incidence was higher in the Rachuonyo district and infections were detected most frequently in 5–10-year-olds. The majority of infections were asymptomatic (58%). Travel away from the study area was a notable risk factor for infection.

**Conclusions:**

Identifying risk factors for malaria infection can help to guide targeting of interventions to populations most likely to be exposed to malaria.

## Introduction

Malaria in African highland areas is typically characterised as unstable or epidemic. Small changes in altitude and temperature, land use and population movement, can result in highly heterogeneous transmission.^1–8^ Highland regions are classified as fringe areas of malaria transmission and are therefore considered possible targets for elimination. Establishing risk factors for infection may help to target interventions to areas or populations where residual transmission remains.

The population residing in the western highlands in Kenya has been subject to malaria epidemics since the 1930s,^9^ despite traditional dogma dictating that the relatively high altitude should have been a barrier to malaria transmission. In more recent years, it appears that malaria in this region has become more stable with a reservoir of asymptomatic malaria infection.^10,11^ The increase in prevalence in the region over the past few decades has generally been attributed to drug failure^9,12^ and a change in mosquito behaviours.^13,14^ In addition, intensification of agricultural production over the past 50 years has changed the entomological landscape of the area^1^ resulting in renewed transmission in areas that were previously malaria-free.

In order to characterise transmission in the region, the Malaria Transmission Consortium (MTC) initiated a programme which consisted of five repeated cross-sectional surveys between 2009 and 2012,^15^ two school surveys in 2009 and 2010,^16^ health facility surveys in 2011 and 2012,^17^ and a cluster randomised trial in 2012.^18,19^ In addition, a cohort study was set up to estimate the incidence of infection in the population. This paper reports on results from the cohort study which ran during 2009 and 2010. The aim of the cohort was to help describe transmission in the area using extended follow-up and to identify risk factors associated with new malaria infections.

## Materials and Methods

### Study site

**Figure 1.**
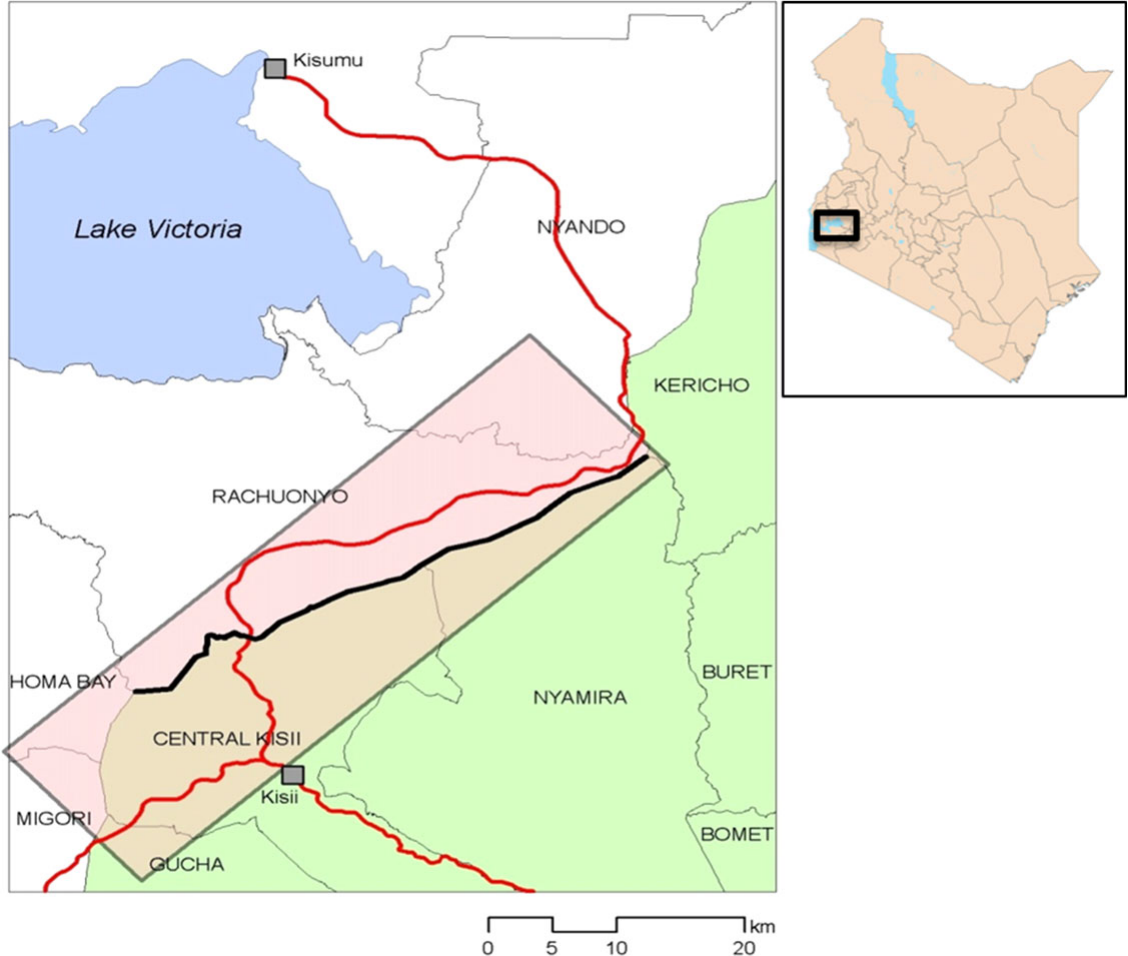
Location of cohort study site within the Kenyan Highlands. Inset: location of the study area within Kenya.

The study site has been previously described in Stevenson et al.,^15^ which summarises results from the baseline cross-sectional study, which took place in June 2009. Briefly, the cohort study took place during 2009 and 2010 in two contiguous districts (Rachuonyo South and Kisii, total population approximately 863 000) close to Lake Victoria in a highland fringe area of Kenya (Figure 1) 1000–1400 m above sea level. Both districts are rural with residents largely dependent upon farming for subsistence. Total annual rainfall averages 1200 mm, while daily temperatures range between 17–27°C. Malaria transmission in the province is relatively high and perennial, with seasonal peaks between April and July, and also November and December.

Insecticide-treated nets (ITNs) have been promoted by the Ministry of Health across both districts, with routine distribution occurring to targeted vulnerable populations through antenatal and child health clinics. Indoor residual spraying (IRS) was implemented in both districts in 2008 using lambda-cyhalothrin capsule suspension (ICON CS; Syngenta, AG, Midrand, South Africa), although coverage varied between districts. *Anopheles arabiensis* and *An. funestus* are the predominant vectors^13^ and *Plasmodium falciparum* is the predominant malaria parasite.

### Study design

The study population was randomly selected from 40 enumeration areas (EAs) which were identified using probability proportional to size; approximately 12 compounds were randomly picked per EA and all inhabitants were asked to take part in the cohort, representing approximately 10% of the population of EAs overall. The cohort study took place for 12 months from May 2009–June 2010. Inclusion criteria for the cohort included living in a selected compound and being aged >6 months. Women who were pregnant at enrolment were excluded from the cohort. At recruitment, all participants were tested using a malaria *P. falciparum-*specific Rapid Diagnostic Test (RDT) (Paracheck, Orchid Biomedical Systems, Goa, India) and given treatment (artemether-lumefantrine, AL) regardless of result in order to ensure all infections were cleared. Baseline data were collected regarding household characteristics such as household assets and construction, travel behaviour, bed net ownership, and use and time since IRS. GPS coordinates were taken for each household.

Follow-up visits occurred every month and involved testing for malaria by RDT and for anaemia using HemoCue (Angelholm, Sweden). Participants found positive by RDT were treated according to national guidelines and those found anaemic were treated with iron. Participants were asked to seek treatment for illness between monthly visits and these data were captured by field workers and incorporated into the final dataset.

Monthly rainfall in mm was collected from one central point in the study area.

### Data management and analysis

Each participant was assigned a unique ID to enable linkage between visits. Data were collected on written forms and double-entered using Microsoft Excel. The dataset was then cleaned and analysed using STATA v.14 (StataCorp LP, College Station, TX, USA).

Primary outcome measures were incidence of *P. falciparum* infection (as determined by PfHRP-2 RDT result) or confirmed test result at the health facility for those individuals who fell ill in between monthly visits, and incidence of moderate/severe anaemia measured using HemoCue, classified by a haemoglobin level <11 g/dl. Altitude was divided into 50 m categories. A socioeconomic status (SES) variable was calculated using Principal Component Analysis (PCA) including household construction and asset ownership variables. The resulting score was divided into quintiles. Chi^2^ tests were used to compare proportions.

Records within two weeks of a RDT-positive visit (and treatment) were removed from the analysis due to the prophylactic effect of AL. Any visits with >6 weeks follow-up time between them were also censored. Total person-time was calculated from date of enrolment to the last visit (minus any long follow-ups or prophylaxis time). Survival analysis was performed using Poisson regression with an exponentiated distribution accounting for clustering at the EA level and multiple failures. Individual risk factors including age, infection status at recruitment and intervention use were investigated in uni- and multi-variable analysis in a forward stepwise manner.

The study was approved by the ethical committees of the London School of Hygiene and Tropical Medicine (protocol #5423) and the Kenya Medical Research Institute (protocol #1517). Meetings were held with district administrative representatives, chiefs and health teams to inform them about the study prior to onset. Community meetings took place to inform potential participants. Individual written consent was obtained from participants >18 years and from those considered mature minors according to national guidelines. Consent for children <13 years of age was provided by a parent/guardian and children aged 13–17 years signed assent forms accompanied by parental/guardian consent.

## Results

Recruitment for the cohort took place during May–June 2009. The characteristics of the study population at recruitment are summarised in Table 1. Briefly, 3155 participants from 974 households (average 3.2 people per household), were followed up as part of the cohort. The median number of visits was 10 and the average time between visits was 32 days. Close to 80% of participants enrolled remained in the cohort at month 12 (78%, 2464/3155). A smaller number of visits took place across the majority of clusters in December 2009 due to population movement for the holidays. Participants were aged 6 months–105 years (median 13 years) and 54% (n=1698) were female. In addition to planned monthly follow-ups, participants with malaria symptoms seeking care between visits were also recorded (termed sick visits). There were 1561 sick visits in total, ranging from 0–7 visits per participant.

**Table 1.** Baseline characteristics of the cohort population

Household characteristics	
Altitude of household (average, range)	1508 m (1437–1628)
Households	974
Households sprayed in the last 12 months	47.5% (463)
SES (household level)	
Highest	27.4% (265)
High	16.1% (156)
Moderate	22.1% (214)
Low	15.0% (145)
Lowest	19.3% (187)
Households with at least one bed net	62.7% (611)
Number of bed nets owned per house (median, range)	2 (1–8)
Households with open eaves	84.9% (827)
Participant characteristics	
People recruited	3155
Age (median, range)	13 years (6 months–105 years)
Sex	
Female	54% (1698)
Male	46% (1454)
Proportion travelling outside of EA within the past 3 months	5.9% (183)
Reporting using a net last night	41.9% (1320)
Anaemic (Hb<11 g/dl)	15.5% (489)
RDT-positive	1.5% (48)

Household wealth varied between the two districts with a higher proportion of households in Rachuonyo classified at the lowest socioeconomic status (28% compared with 11% in Kisii, p<0.001). Approximately 42% (n=1320) of participants reported using a net the previous night and 48% of households reported spraying within the previous 12 months. Due to the programmatic design of the IRS campaign, which aimed for blanket coverage of certain higher prevalence districts and targeted spraying in others, more households had been sprayed in Rachuonyo within the last 12 months (73% compared with 23% in Kisii).

At baseline, 12% (n=372) of participants reported having a fever within the previous month. Reported fevers were most common in people aged >20 years (16% reported fever compared with 7% of children aged 10–15 years). Current temperature was not recorded at the baseline visit. Also, 489 (15.5%) of participants were anaemic, with prevalence highest in children aged <5 years (37%). Forty-eight (1.5%) of participants were RDT-positive at baseline. Seventeen (35%) of RDT-positive individuals were anaemic. Forty (83%) of the malaria infections were in children aged ≤15 years.

### Incidence of *P. falciparum* infection

**Figure 2:**
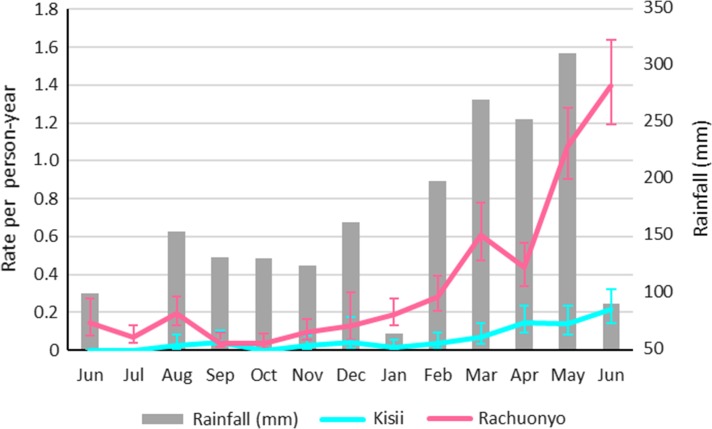
Incidence per month in each district (95% CI) and rainfall in the study area.

During the 12 months of follow-up time, 621 *P. falciparum* infections were detected in 500 participants (16%) (either during follow-up visits or through presentation to clinic), the equivalent of 0.2 cases per person. The average number of days to first infection was 283 (32–383). A larger number of infections was detected in Rachuonyo (n=539) compared with Kisii (n=82). The highest number of infections was detected in the final month of the cohort study (June 2010) (n=177), with the increase appearing closely correlated to an increase in rainfall across the study area (Figure 2).

The highest proportion of RDT-detectable infections was in children aged 5–10 years, where one-fifth of participants experienced an infection (21%, 124/481). Infections were generally more common in younger participants; 18% of children aged ≤15 years had an infection compared with 13% of participants aged >15 years (p=0.001).

Three hundred and sixty-two infections (58%) were classified as asymptomatic (no fever at time of visit or within 48 h before). The highest proportion of asymptomatic infections were in those aged 15–20 years where 68% of infections were asymptomatic, compared with 52% in those aged <5 years. Anaemia was much more prevalent in those aged <5 years, where it was detected at 29% of visits (n=1888), compared with just 5% (n=223) in those aged 10–15 years.

### Risk factors for *P. falciparum* infection

The incidence rate (per 1000 people) and hazard ratio (HR) was calculated for each possible risk factor for infection (Table 2). There was no evidence for a difference in infection risk for sex or altitude band. The highest infection rate was detected in the youngest age group (0–5 years) with the lowest in those aged 25–50 years. There was a substantially higher risk [HR 2.6 (1.8–3.7), p<0.001] in individuals living in households that had been sprayed within the last 12 months; however, this was likely due to the stratified IRS campaign which preferentially targeted higher risk areas. Net use appeared to have no effect on risk. People who had recently travelled were more likely to be infected [HR 1.7 (1.1–2.7), p=0.045]. In addition, people who were infected at recruitment were more likely to have a subsequent infection [HR 2.4 (1.6–3.5), p<0.001]. When all other risk factors were accounted for, age, SES, travel outside of EA and infection status at recruitment remained risk factors for infection, with people who had travelled nearly twice as likely to be infected [HR 1.8 (1.1–3.0), p=0.02] and people who were infected at recruitment nearly three times as likely to get a subsequent infection [HR 2.6 (1.7–4.1), p<0.001] (Table 3).

**Table 2.** Characteristics of the population who experienced a malaria infection

	Number of cases	Follow-up time (years)	Incidence rate (per 1000 person-years)	Hazard ratio (95% CI)	P value
Sex					
Female	315	1579	199.4	1.0	0.156
Male	306	1366	224.1	1.1 (0.9–1.3)	
Age group (years)					
0–5	134	571	234.6	1.0	0.006
5–15	228	568	225.2	1.1 (0.8–1.4)	
15–25	63	443	162.2	0.8 (0.6–1.2)	
25–50	78	232	140.5	0.6 (0.4–1.0)	
50–100	59	104	180.9	0.7 (0.5–0.9)	
Socioeconomic status					
Highest	100	703	142.2	1.0	0.026
High	67	465	144.2	1.0 (0.6–1.6)	
Medium	129	664	194.4	1.4 (0.9–1.9)	
Lower	103	451	228.4	1.6 (1.0–2.6)	
Lowest	159	570	278.7	1.9 (1.2–3.2)	
Altitude (m)					
1437–1488	139	735	188.9	1.0	0.618
1489–1508	159	737	215.8	1.1 (0.9–1.4	
1509–1526	129	676	190.8	1.0 (0.8–1.3)	
1527–1628	137	714	191.96	1.0 (0.8–1.3)	
Spray status					
Not sprayed in last 12 months	155	145	107.1	1.0	<0.001
Sprayed in last 12 months	413	143	288.9	2.7 (1.8–3.9)	
Net use					
Did not use a net	336	168	200.3	1.0	0.742
Used a net	232	119	193.4	0.9 (0.8–1.2)	
Travel outside of EA					
No	508	2681	189.5	1.0	0.045
Yes	52	17	313.7	1.7 (1.0– 2.7)	
Infection status at recruitment					
Not infected	545	2830	192.6	1.0	<0.001
Infected	21	45	468.7	2.4 (1.6–3.7)

**Table 3.** Results from adjusted multi-variable Poisson regression

Covariate	Category	Adjusted hazard ratio	P value
Age group (years)	0–5	1.0	0.011
	5–10	1.1 (0.9–1.5)	
	10–15	0.9 (0.6–1.2)	
	15–20	0.7 (0.5–1.1)	
	>20	0.7 (0.5–0.9)	
SES	Highest	1.0	0.016
	High	1.1 (0.7–1.8)	
	Medium	1.5 (1.0–2.1)	
	Lower	1.8 (1.2–2.7)	
	Lowest	2.1 (1.3–3.3)	
Travel outside of the EA	No	1.0	0.024
	Yes	1.8 (1.1–3.0)	
Infection status at recruitment	Not infected	1.0	<0.001
	Infected	2.6 (1.7–4.1)	

### Spatial and temporal clustering of infection

**Figure 3.**
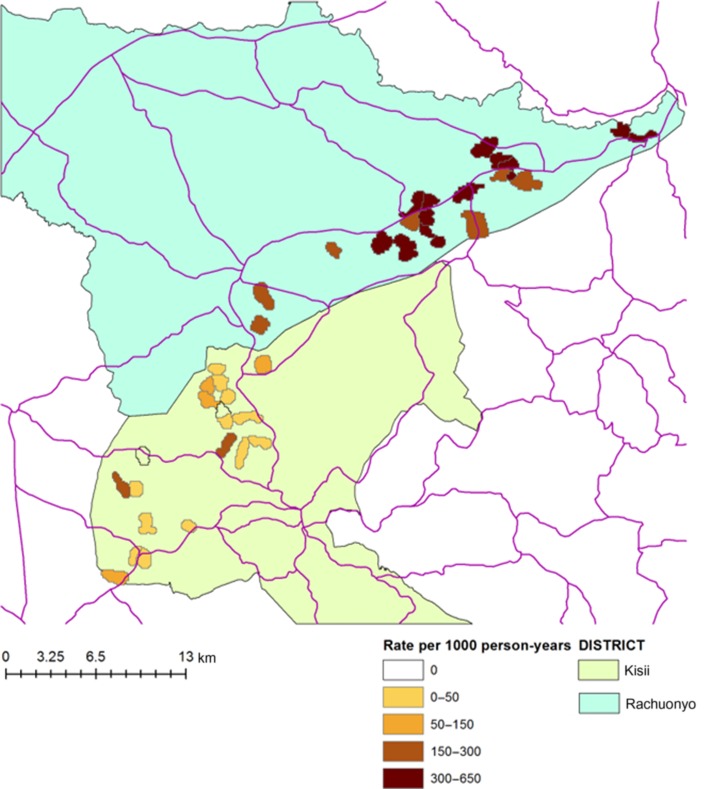
Infection incidence per 1000 person-years in the 40 cohort study clusters.

Incidence was higher in Rachuonyo compared with Kisii (Figure 3 and supplementary video). People who were infected at enrolment were substantially more likely to be infected during follow-up than those who were not infected at enrolment. While only 16% of cohort participants (n=500) experienced infections, many were infected more than once. Three people experienced 4 infections during the follow-up year, all aged <10 years and living in Rachuonyo. Infections were clustered within households, with 70% of households experiencing no infections during the cohort study; 89% of households in Kisii experienced no infections compared with 51% of households in Rachuonyo. Nearly 75% of infections (n=407) occurred in the final 4 months of the cohort study (March–June 2010) (Figure 2), with 86% (n=351) of those occurring in Rachuonyo.

## Discussion

As transmission reduces and becomes more heterogeneous,^20,21^ malaria surveillance systems need to switch from facility-based aggregated data to focus on individual infections and risk factors. Cohort studies are one way to identify potential risk factors for ongoing transmission in a community. This cohort study in a highland area of western Kenya highlights temporal and spatial differences in *P. falciparum* incidence risk over relatively short times and distances, with repeat infections among individuals and substantial clustering within households.

The study took place at a median altitude of 1500 m above sea level. High altitude has a notable impact on malaria transmission^22,23^ due to lower temperatures increasing the time required for sporogeny within the mosquito, which consequently reduces the likelihood of transmission cycle completion as mosquitoes may not live long enough to permit parasite development. Previous studies in the Kenyan Highlands have detected differences in malaria prevalence over relatively small changes in altitude,^24^ with Githeko et al. describing a 16% reduction in prevalence for every 50 m increase in altitude, and Baidjoe et al. reporting prevalence variations between areas with as little as a 10 m difference in altitude.^25,26^ In this cohort study, as with the baseline cross-sectional survey which took place in the same clusters,^15^ no difference in parasite measures was detected within the relatively small range of altitude of the study site. Serological measures in the same clusters, however, indicated that altitude may have had an impact on longer-term malaria exposure, with lower seroprevalence detected in the highest altitude bands.^26^

Overnight travel away from home was a notable risk factor for higher *P. falciparum* incidence. Although highland areas have potentially lower receptivity to malaria due to cooler temperatures, vulnerability to imported infections is a risk due to regular population movement to areas of lower altitude (and consequently potentially higher endemicity) for commercial purposes. Travel has been noted as a risk factor in other highland areas of Kenya^4,27,28^ and elsewhere in East Africa.^29^ In the baseline cross-sectional survey, travel was not found to be associated with higher infection prevalence,^15^ which the authors hypothesised was due to the majority of travel potentially being to non-endemic Nairobi. The identification of risk factors is potentially more precise using incident measures, which detect new infections, as compared with prevalence, which identifies ongoing infections, which could have been present for a longer time period and as a result of other risk behaviours. In addition, the longitudinal nature of a cohort study means further nuance may be detected using risk factor analysis. The importance of travel as a risk factor within our cohort may have been exacerbated by the so-called ‘cohort effect’. Our cohort population represented approximately 10% of the population within the study area and the increased surveillance (and subsequent treatment) within this group may have resulted in an overall transmission reduction in the area.

Intervention use did not appear to reduce infection in this cohort. IRS targeted to high prevalence areas took place during the study period which meant that living in a sprayed household was associated with infection. Also, use of bed nets did not appear to be protective, which is in contrast to many studies showing the use of bed nets to be protective against infection (reviewed in^30,31^). We suspect that the lack of protection in this cohort is due to the use of bed nets being based on a question asked at enrolment, rather than during each follow-up visit. While use of a net the night before enrolment may be a useful proxy, perhaps in this study it was not representative of actual net use, which is known to be seasonal in some settings. Another reason for the lack of apparent association with protection could be due to people not using bed nets when they travel, which was a clear risk factor in this cohort. Educational campaigns encouraging net use have taken place across the study area to increase usage. One area of focus could be in encouraging the use of interventions during travel. A subsequent study looking at the combination of IRS and ITNs in the Rachuonyo district found that using a net and having IRS provided significantly greater protection against infection than just using ITNs alone.^32^

This cohort study was one aspect of a larger study run by the MTC looking at characterising malaria transmission in the western Kenyan Highlands. Despite being resource-intensive, cohort studies have the advantage of measuring the risk of contracting new infections, which can help to more precisely identify risk factors, compared with looking at prevalence of infection within the community. In addition, cohorts can assess seasonal fluctuations in transmission (shown to be substantial in this area), which can assist with planning timings for efficient vector control. Cohort studies work best in stable populations and are not always ideal in highland areas where population movement can be high; however, loss to follow-up in this study was minimal.

The relationship between prevalence and incidence is complex.^33–35^ Typically, programmes measure malaria incidence through passive detection at health facilities, and as such only include symptomatic infections that occur in the healthcare-seeking population. In areas of moderate to high transmission, exposure-based immunity is likely to mean that passive detection will predominantly only include cases in children. Actively measured incidence (i.e. testing people regardless of symptoms), such as occurred in this cohort study, is likely to generate results that more accurately reflect the transmission of malaria in a population. However, infections in previously exposed individuals may potentially be of lower parasite density and could remain undetected by the diagnostic used in this study, RDT. In the baseline cross-sectional survey, 65% of all infections were only detectable by molecular methods,^15^ which suggests there is a substantial reservoir of low-density infection in the population. Potentially, further risk factors may have been identified within the cohort if low-density infections had also been detected, and the age distribution of infections may also shift.

Malaria infections were highly clustered temporally and spatially. Little difference in incidence was evident between the two districts until rainfall increased from March onwards when rains began, at which point the population in Rachuonyo experienced much higher incidence than those living in Kisii. Rainfall increases the availability of mosquito-breeding sites, which can be very focal depending on the topology of the area, with breeding sites often being confined to lower altitudes where drainage is less efficient.^25^ In another highland site in Kenya, the highly heterogeneous distribution of vectors led to large differences in infection prevalence between sites only a few km apart,^8^ and drainage has also been indicated as a cause of heterogeneous transmission in other areas in Kenya.^10^ Despite only being separated by a road, the difference in transmission between the two districts was stark and may be related to different topologies.

The diagnostic used in this study presents some limitations. It only detects *P. falciparum* infection, which although the main Plasmodium species in the area, means that we did not assess risk factors associated with the transmission of the other potential species present, *P. malariae* and *P. ovale*. In addition, the RDT is also likely to have missed a proportion of the low-density infections present in the population, as well as potentially missing parasites with HRP-2/3 deletions.

## Conclusion

Numerous surveys have taken place within this region of the Kenyan Highlands in order to characterise transmission and identify risk factors in an area where transmission is known to be heterogeneous and unpredictable. Transmission in this cohort was remarkably clustered, both temporally and spatially, with the majority of the population not experiencing any exposure. Identifying those that remain at risk of infection, such as those who experienced repeated infections, can help to guide more targeted, cost-efficient intervention approaches. Net use was relatively low with the highest risk group (school-age children) being least likely to report using a net. At this low usage, the added mass effect when net use is high, will not be achieved. Increasing net use within populations living in high-risk geographical areas, such as the Rachuonyo district, and within populations at higher risk, such as people travelling overnight to higher endemic areas, could help further reduce transmission in the region.

## Supplementary Material

Supplementary Data
